# High-titer 2-phenylethanol production in *Escherichia coli* via decarboxylation-step optimization and a vegetable-oil overlay for biocompatible in situ recovery

**DOI:** 10.1186/s13036-026-00667-4

**Published:** 2026-03-27

**Authors:** Chang Liu, Hongwei Zhang, Wanbin Xing, Rina Na, Xuanyu Meng, Sichen Huang, Linlin Wang, Guoqiang Cao, Pengchao Wang

**Affiliations:** 1 State Key Laboratory of Utilization of Woody Oil Resource, Northeast Forestry University, Harbin, Heilongjiang, 150040 China; 2 School of Life Science, Northeast Forestry University, Harbin, Heilongjiang, 150040 China; 3 Aulin College, Northeast Forestry University, Harbin, Heilongjiang, 150040 China; 4 College of Wildlife and Protected Area, Northeast Forestry University, Harbin, Heilongjiang, 150040 China; 5 Xiangbai Biotechnology Co., Ltd., Harbin, Heilongjiang, 150040 China

**Keywords:** *Escherichia coli*, 2-phenylethanol, Decarboxylation, In situ product recovery, Metabolic engineering, Aromatic alcohols

## Abstract

**Background:**

2-phenylethanol (2-PE) is a high-value aromatic alcohol. Its bioproduction is limited by pathway bottlenecks, limited by a rate-limiting decarboxylation step, product toxicity, and volatilization. We sought to build an efficient *Escherichia coli* (*E. coli*) platform by optimizing the segment of the Ehrlich route that converts L-phenylalanine (L-Phe) to 2-PE, together with a food-grade soybean-oil overlay for in situ product recovery (ISPR).

**Results:**

Whole-cell assays showed that phenylpyruvate decarboxylation was the main bottleneck when the yeast decarboxylase Aro10 was used. A phylogeny-guided screen identified Lactococcus lactis KivD as a superior substitute in *E. coli*. After replacing Aro10 with KivD and tuning expression, leveraging endogenous glutamate dehydrogenase (GDH) for NAD(P)H recycling enabled sufficient cofactor regeneration, yielding 49.5 mM 2-PE from 50 mM L-Phe with 99.0% conversion. At the shake-flask scale, a fed-batch L-Phe feeding strategy coupled with a 2:1 soybean-oil overlay for in situ product removal reduced toxicity and volatilization losses, producing 130 mM 2-PE (15.9 g/L). Using soybean oil as an ISPR phase effectively sequestered 2-PE from the aqueous phase, minimizing volatilization and increasing the overall recovered yield to 94.2% based on L-Phe consumption.

**Conclusions:**

Replacing the rate-limiting decarboxylation step with KivD, balancing redox through cofactor recycling, and coupling the pathway to a mild oil-overlay recovery increased flux and enabled high-titer 2-PE in *E. coli*. The workflow of bottleneck substitution plus biocompatible in situ recovery can be transferred to related aromatic alcohols and provides a foundation for de novo routes and high-cell-density fermentations that target still higher titers.

**Graphical Abstract:**

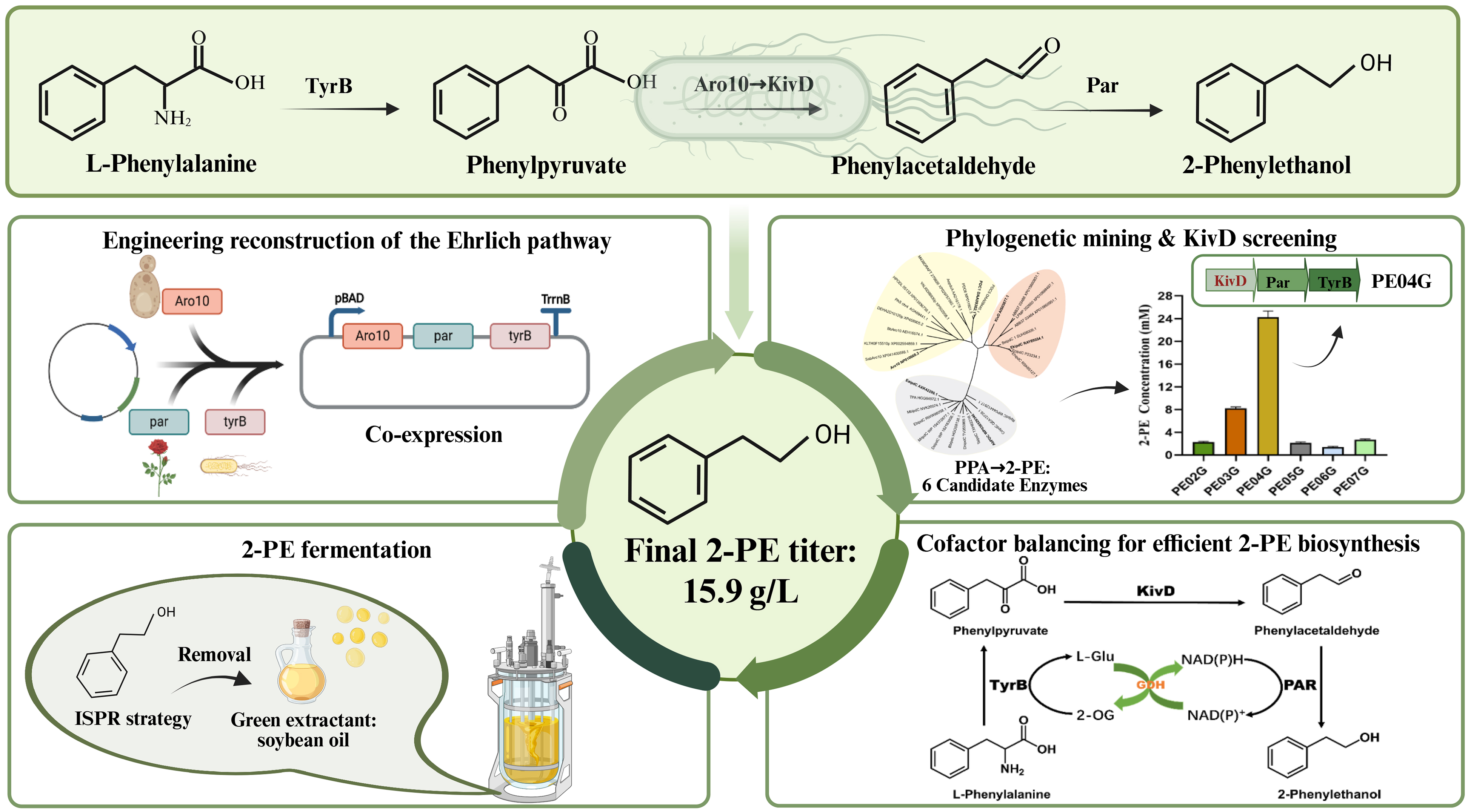

**Supplementary Information:**

The online version contains supplementary material available at 10.1186/s13036-026-00667-4.

## Background

2-PE is a naturally occurring aromatic alcohol characterized by an elegant, delicate, and long-lasting rose-like fragrance [1–3]. As a natural compound found in plant sources such as roses, grapes, and green tea [4–6], 2-PE is regarded as a potentially more sustainable, renewable, and environmentally friendly option compared to certain synthetic fragrances [7], aligning with the growing demand for comparatively eco-friendly and safe additives in modern consumer products [8–10].

Owing to its unique rose-honey aroma, low toxicity, and excellent dermal safety, 2-PE has found broad applications in the personal care, food, and pharmaceutical industries [11–13]. In personal care products, 2-PE is commonly added to cosmetics and household cleaning products due to its non-toxic nature and pleasant scent, and perfumes as a non-toxic fragrance ingredient [14–16]. In foods, 2-PE is a widely used flavor additive contributing to the aroma and palatability of beverages and processed products [17–21]. In pharmaceuticals and related applications, it functions as an antimicrobial preservative and a precursor to high-value derivatives [22–27]. Driven by the clean label movement and consumer preference for natural and perceived sustainable products, market demand for 2-PE continues to rise, with projections of a 5–6% CAGR from 2021 to 2027 and a market size exceeding USD 350 million by 2027 [28–32].

Currently, industrial production of 2-PE mainly relies on plant extraction or chemical synthesis. In plants, 2-PE can be recovered from flowers or tissues via extraction and concentration, typically following steam distillation to obtain essential oils and subsequent purification [33, 34]. Although plant-extracted 2-PE offers high purity, non-toxicity, and excellent safety profiles, the purification process is labor-intensive and costly—reaching up to USD 1000 per kilogram—while the yield remains low and fails to meet commercial demand [35, 36]. Therefore, most 2-PE on the market is produced via petrochemical routes, such as Friedel–Crafts alkylation, catalytic hydrogenation of styrene, or recovery as a by-product during styrene production [37–39], with substantially lower costs (approximately USD 5 per kilogram) [1, 5]. Nevertheless, these routes face sustainability concerns, including reliance on petroleum-derived feedstocks [40, 41], generation of corrosive and toxic by-products and hard-to-remove impurities that may restrict applications in food and personal care products [7, 11], and energy-intensive operations associated with pollutant emissions and environmental impacts [42–44].

Consequently, microbial biosynthesis based on renewable feedstocks has emerged as a promising strategy for the potentially more sustainable production of 2-PE. Studies have shown that certain eukaryotic organisms, such as *Saccharomyces cerevisiae*, *Yarrowia lipolytica*, *Pichia pastoris*, and *Aspergillus oryzae*, are capable of naturally producing 2-PE, providing a biological basis for fermentation-based production processes [45, 46] Compared with traditional chemical synthesis, microbial fermentation may better satisfy specific green chemistry criteria by using renewable substrates, operating under mild and low-energy conditions, and avoiding toxic organic solvents, thereby reducing waste and emissions [47, 48]. Moreover, microbial catalysis typically often provides high product selectivity with fewer undesired by-products and can be implemented on diverse renewable feedstocks, improving atom economy and reducing the formation of undesired by-products [49]. When produced from eligible natural sources in compliance with relevant regulatory definitions, fermentation-derived 2-PE may be marketed as a “natural flavor,” improving regulatory and market acceptance [6, 50]. Therefore, microbial biosynthesis represents a feasible option with potential sustainability advantages for the industrial-scale production of 2-PE.

To date, three microbial biosynthetic pathways for 2-PE production have been reported: the Ehrlich pathway, the styrene-derived pathway, and the phenylethylamine pathway [51]. The Ehrlich pathway is the most widely employed, converting L-Phe to 2-PE via transamination, decarboxylation, and reduction, and has been implemented in yeasts such as *Saccharomyces cerevisiae* and *Yarrowia lipolytica* [52–55]. It is attractive for industrial applications due to its low-cost substrates and well-defined steps, and can also valorize agro-industrial by-products, including molasses, grape pomace, whey, tobacco waste, lignocellulosic hydrolysates, and biodiesel-derived crude glycerol [56–60]. Therefore, the Ehrlich pathway holds strong potential for integration with renewable resources to support more environmentally friendly 2-PE production [61, 62]. However, 2-PE is inherently toxic to microbial hosts, as it disrupts membrane integrity and reduces cell viability, which severely limits its accumulation during fermentation [63, 64]. Accordingly, strain adaptation, metabolic rewiring, and ISPR have been developed; for example, adaptive evolution and pathway optimization in S. cerevisiae enabled 3.14 g/L 2-PE from L-Phe [65]. In addition, in situ product adsorption using macroporous resins or hydrophobic PMMA microspheres has been applied in *S. cerevisiae* and *Acinetobacter soli* fermentations, effectively removing 2-PE from the culture broth and increasing titers to approximately 6–7 g/L [66–68]. Biphasic ISPR overlays further improved production: oleic acid increased the total titer from 4.81 to 6.41 g/L in a 5 L fermentation [69], polypropylene glycol (PPG) 1200 raised the titer from 0.9 to 10.2 g/L [70]. In addition, a biphasic system with rapeseed oil as a food-grade extractant has achieved 9.79 g/L 2-PE while simultaneously generating a rose-scented oil co-product [60].

In our previous work, a cofactor self-sufficient *E. coli* whole-cell biocatalyst was established, in which GDH simultaneously regenerated 2-oxoglutarate (2-OG) and NAD(P)H and zeolite was used to remove ammonium, achieving the highest 2-PE titer then reported for an *E. coli* Ehrlich-type system from L-Phe [71]. However, although the GDH-based bridge was designed to recycle 2-OG and NAD(P)H in a stoichiometric manner, quantitative conversion was not achieved, suggesting a kinetic mismatch between cofactor recycling and the Ehrlich module. This motivated us to pinpoint the rate-limiting step that decouples cofactor balance from product formation. Building on this platform, the present study advances beyond cofactor self-sufficiency by targeting two remaining constraints: the rate-limiting phenylpyruvate decarboxylation step and 2-PE toxicity during production. Specifically, decarboxylation was identified as the bottleneck; a highly active phenylpyruvate decarboxylase (PDC) was selected via phylogenetic analysis and its performance was further improved through promoter engineering. The GDH-based cofactor self-cycling module was retained to sustain NAD(P)H supply and support downstream reduction once decarboxylation was accelerated. To mitigate product inhibition, we developed biphasic ISPR systems and highlight edible vegetable oils as food-grade overlays, offering a practical alternative to oleic acid or polypropylene glycol. Collectively, this work establishes an efficient and scalable microbial platform toward more sustainable 2-PE production.

## Materials and methods

### Chemicals and standards

L-Phe (≥ 99% purity) was purchased from Shanghai Yuanye Bio-Technology Co., Ltd. Analytical-grade 2-PE, PPA, and PAld, each with a purity of ≥ 99%, were obtained from Shanghai Aladdin Biochemical Technology Co., Ltd. HPLC-grade acetonitrile, methanol, and formic acid were purchased from Shanghai Aladdin Biochemical Technology Co., Ltd. Oleic acid and PPG1000 used as extraction phases were of reagent grade and purchased from Shanghai Aladdin Biochemical Technology Co., Ltd. Food-grade refined soybean oil (Jinlongyu, China) was purchased locally. All other chemicals and reagents used in this study were standard laboratory consumables for molecular biology applications and were handled according to the manufacturers’ instructions.

### Strains and plasmid construction

Chemically competent *E. coli* DH5α and BW25113 strains used in this study were purchased from Shanghai Weidi Biotechnology Co., Ltd. The BW-Δgdh strain (a gdhA-deficient mutant derived from *E. coli* BW25113) was constructed in our laboratory as described previously [71] and preserved in our laboratory. The plasmids pYB1a and pY97a used in this work were also maintained in-house.

All plasmids were constructed using Gibson assembly with the Gibson Assembly Cloning Kit (New England Biolabs, USA), following the manufacturer’s protocol. Plasmid assemblies were verified by colony PCR, restriction digestion, and Sanger sequencing. Successfully assembled plasmids were introduced into competent *E. coli* cells via the standard heat-shock transformation method. Transformants were selected on LB agar plates supplemented with ampicillin (100 mg/L), and ampicillin selection was maintained during cultivation where appropriate. Details of the engineered strains and their corresponding genetic modifications are provided in the Supplementary material (Table S1).

### Cultivation and induction of engineered strains

A single colony from an LB agar plate was inoculated into 5 mL of liquid LB medium supplemented with 100 mg/L ampicillin and cultured overnight at 37 °C with shaking at 200 rpm. Subsequently, 1 mL of the overnight culture was transferred into 100 mL of ZYM-5052 medium supplemented with ampicillin (100 mg/L) and incubated at 37 °C and 200 rpm for 1–2 h. When the optical density at 600 nm (OD₆₀₀) reached 0.4–0.8, L-arabinose was added to a final concentration of 0.2% (w/v) to induce protein expression. The temperature was then reduced to 30 °C for induction unless otherwise specified, and shaking incubation was continued at 200 rpm until OD₆₀₀ reached 8–10 for subsequent whole-cell biocatalysis experiments.

The composition of ZYM-5052 medium was as follows: 10 g/L tryptone, 5 g/L yeast extract, 50 mM phosphate buffer, 50 mM ammonium chloride, 5 mM sodium sulfate, 0.5% glycerol, 0.05% glucose, and 0.2 mM magnesium sulfate.

### SDS-PAGE analysis

Approximately 6 OD₆₀₀ units (equivalent to 6 mL of culture at OD₆₀₀ = 1) of the induced *E. coli* culture were harvested in 1.5 mL microcentrifuge tubes and centrifuged at 4200 rpm (~ 1,200 ×g) for 10 min. The supernatant was discarded, and the cell pellet was resuspended in 600 µL of sterile water. Cell disruption was performed using an ultrasonic homogenizer with a 2 mm microtip on ice (30% output; pulse 5 s on/5 s off; total sonication time 5 min) to obtain crude protein extracts. The lysate was centrifuged at 10,000 ×g for 10 min to separate the soluble and insoluble protein fractions. The supernatant (soluble protein) was collected, while the pellet (insoluble fraction) was resuspended in 600 µL of sterile water. Equal volumes of each fraction were mixed with 5× protein loading buffer and denatured at 95–100 °C for 5 min. SDS-PAGE was performed using self-prepared polyacrylamide gels with a 5% stacking gel and a 12% separating gel. Electrophoresis was stopped when the loading dye front approached the bottom of the gel. The gel was stained with Coomassie Brilliant Blue for 1 h and destained until clear protein bands were visible.

### Whole-cell biocatalysis

#### Small-scale whole-cell biocatalysis

A 50 mM Tris-HCl buffer was prepared and adjusted to pH 7.0 unless otherwise specified using concentrated hydrochloric acid. L-Phe powder was dissolved in the buffer to a final concentration of 50 mM, with shaking to ensure complete dissolution, yielding the reaction mixture. The OD_600_ of the induced culture was measured at 600 nm using a UV-Vis spectrophotometer, with uninoculated ZYM-5052 medium as the blank. Cells equivalent to 6 OD_600_ units were harvested by centrifugation at 4200 rpm (~ 1,200 ×g) for 10 min at 4 °C, where 1 OD unit is defined as 1 mL of culture at OD_600_ = 1. Cell dry weight (DCW; g/L) was estimated from OD_600_ using a published conversion factor for *E. coli* (1 OD_600_ ≈ 0.3 g DCW/L) [72]. The pellet was washed once with 1 mL of 1× PBS (pH 7.4) and centrifuged again to remove the supernatant. The cells were then resuspended in 200 µL of the prepared reaction mixture (corresponding to a final cell density of 30 OD units/mL, ≈ 9.0 g DCW/L, estimated) and transferred to 1.5 mL microcentrifuge tubes. The whole-cell biotransformation was carried out at 30 °C with shaking at 200 rpm. Microcentrifuge-tube reactions were incubated for 12 h before analysis.

#### Flask-scale whole-cell biocatalysis

For larger-scale reactions, 2000 OD_600_ units of the induced culture were collected by centrifugation at 4200 rpm (~ 1,200 ×g) for 10 min at 4 °C. After discarding the supernatant, the cells were washed once with 50 mL of 1× PBS (pH 7.4) and recentrifuged. The pellet was resuspended in 20 mL of the reaction mixture (corresponding to a final cell density of 100 OD units/mL, ≈ 30 g DCW/L, estimated) and transferred into 250 mL Erlenmeyer flasks. Whole-cell biocatalysis was performed at 28.7 °C, as determined by response surface methodology, with shaking at 200 rpm, and samples were taken at specified time intervals for subsequent analysis. Residual L-Phe was measured by offline High-performance liquid chromatography (HPLC) every 2 h. Feeding was adaptive based on these measurements rather than a predetermined schedule: when residual L-Phe in the aqueous phase dropped below 10 mM, solid L-Phe was added to restore the aqueous-phase concentration to 50 mM. The interval between feeding events was typically 6 h; no further feeding was performed in the late phase as the conversion rate gradually decreased.

### Quantification of 2-PE

HPLC was employed to quantify L-Phe and 2-PE in the reaction mixtures. A small aliquot of the reaction solution was collected and diluted 50–100-fold as needed to keep peak areas within the linear range of the standard calibration curves. The sample was centrifuged at 10,000 ×g for 10 min, and the supernatant was filtered through a 0.22 μm sterile membrane filter into HPLC vials for analysis. HPLC analysis was performed on an Agilent LC1100 system equipped with a Welch XB-C18 column (4.6 × 250 mm, 5 μm). The mobile phase consisted of acetonitrile, water, and formic acid in a volume ratio of 30:70:0.5 (v/v/v). The detection wavelength was set at 214 nm, with a flow rate of 1.0 mL/min, an injection volume of 10 µL, and a constant column temperature of 30 °C. The total run time was 12 min. The HPLC chromatograms of L-Phe and 2-PE standards, as well as their corresponding standard calibration curves, are provided in the Supplementary material (Figure S1).

### Phylogenetic tree construction

To identify candidate PDCs for phenylpyruvate (PPA) decarboxylation, a phylogenetic analysis was performed based on amino acid sequences of PDC candidates from various organisms. A total of 33 protein sequences were retrieved from the NCBI database using “phenylpyruvate decarboxylase” as the keyword. Multiple sequence alignment was conducted using the ClustalW algorithm, and a phylogenetic tree was constructed in MEGA 11 (version 11.0.13) using the Neighbor-Joining (NJ) method. Genetic distances were calculated using the p-distance model, and the reliability of the tree topology was evaluated by bootstrap analysis with 1000 replicates.

### Response surface methodology design

Response surface methodology was employed using Design-Expert software (version 13.0.15) to optimize the induction and whole-cell biocatalysis conditions for enhanced 2-PE production. A three-factor, three-level Box–Behnken Design (BBD) was adopted, with the following independent variables: induction temperature (X₁), whole-cell biocatalysis temperature (X₂), and reaction pH (X₃). The ranges of the variables were set as follows: induction temperature from 15 °C to 40 °C, whole-cell biocatalysis temperature from 15 °C to 40 °C, and pH from 5.0 to 9.0. The response variable (Y) was defined as the 2-PE titer quantified by HPLC under each experimental condition. A total of 20 experimental runs were designed for model construction and optimization. Regression analysis and analysis of variance (ANOVA) were applied to assess the model’s goodness of fit and the statistical significance of each factor. Response surface plots illustrating the interactions among X₁, X₂, and X₃ are provided in the Supplementary material (Figure S5).

### Evaluation of 2-PE volatility under whole-cell biocatalysis conditions

To assess the natural volatilization of 2-PE under whole-cell biocatalysis conditions, a defined amount of 2-PE was diluted with 50 mM Tris-HCl buffer to a final volume of 20 mL, yielding a concentration of 50 mM. The solution was transferred to a 250 mL Erlenmeyer flask and incubated at 28.7 °C with shaking at 200 rpm without cells and without an oil overlay. The flask was sealed with a breathable film. Samples were taken every 2 h, diluted 50-fold, and analyzed by HPLC as described above to quantify residual 2-PE concentrations over time and evaluate volatility-related losses.

### In situ extraction method

To minimize product volatilization and alleviate product inhibition during whole-cell biocatalysis in shake flasks, an in situ extraction strategy was applied using oleic acid or food-grade refined soybean oil as the organic extraction phase. The oil phase and aqueous phase were mixed at a volume ratio of either 1:1 or 2:1 (v/v). The organic phase was added at the start of flask-scale whole-cell biocatalysis. During sampling, the flask was allowed to stand for approximately 1 min to facilitate phase separation. Samples were then collected separately from the aqueous and oil phases by carefully withdrawing from the middle of each layer with a pipette while avoiding the interphase. Aqueous samples were diluted with double-distilled water, while oil-phase samples were diluted with pure acetonitrile or methanol. All samples were filtered through 0.22 μm syringe filters and analyzed by HPLC.

To calculate productivity metrics, the total 2-PE titer (mM) was converted to g/L using the molecular weight of 2-PE (MW = 122.17 g/mol): *C*_*g/L*_ *= C*_*mM*_ *× MW/1000*. The average volumetric productivity was calculated as *P*_*V*_ *= C*_*g/L*_*/t* (g 2-PE/L·h), where *t* is the reaction time. The average specific productivity was calculated as *P*_*S*_ *= P*_*V*_*/X* (g 2-PE/g DCW·h), where *X* is the biomass concentration (g DCW/L) estimated from OD_600_ as described above. For biphasic reactions, the oil-to-aqueous volume ratio was defined as *n = V*_*oil*_*/V*_*aq*_. The total 2-PE titer was calculated on an aqueous-volume basis by summing the aqueous- and oil-phase contributions: *C*_*2−PE, total*_ *= C*_*2−PE, aq*_ *+ n*⋅*C*_*2−PE, oil*_ (mM), where *C*_*2−PE, aq*_ and *C*_*2−PE, oil*_ are the concentrations quantified in the aqueous and oil phases, respectively. The overall recovery (recovery efficiency) was calculated relative to the theoretical 2-PE estimated from L-Phe consumption assuming a 1:1 stoichiometry: *Recovery (%) = C*_*2−PE, total*_*/ΔC*_*L−Phe*_*×100*, where *ΔC*_*L−Phe*_ is the apparent L-Phe consumption determined from the decrease in aqueous-phase L-Phe concentration.

## Results

### Construction of a 2-PE biosynthetic pathway and identification of the catalytic bottleneck

**Fig. 1:**
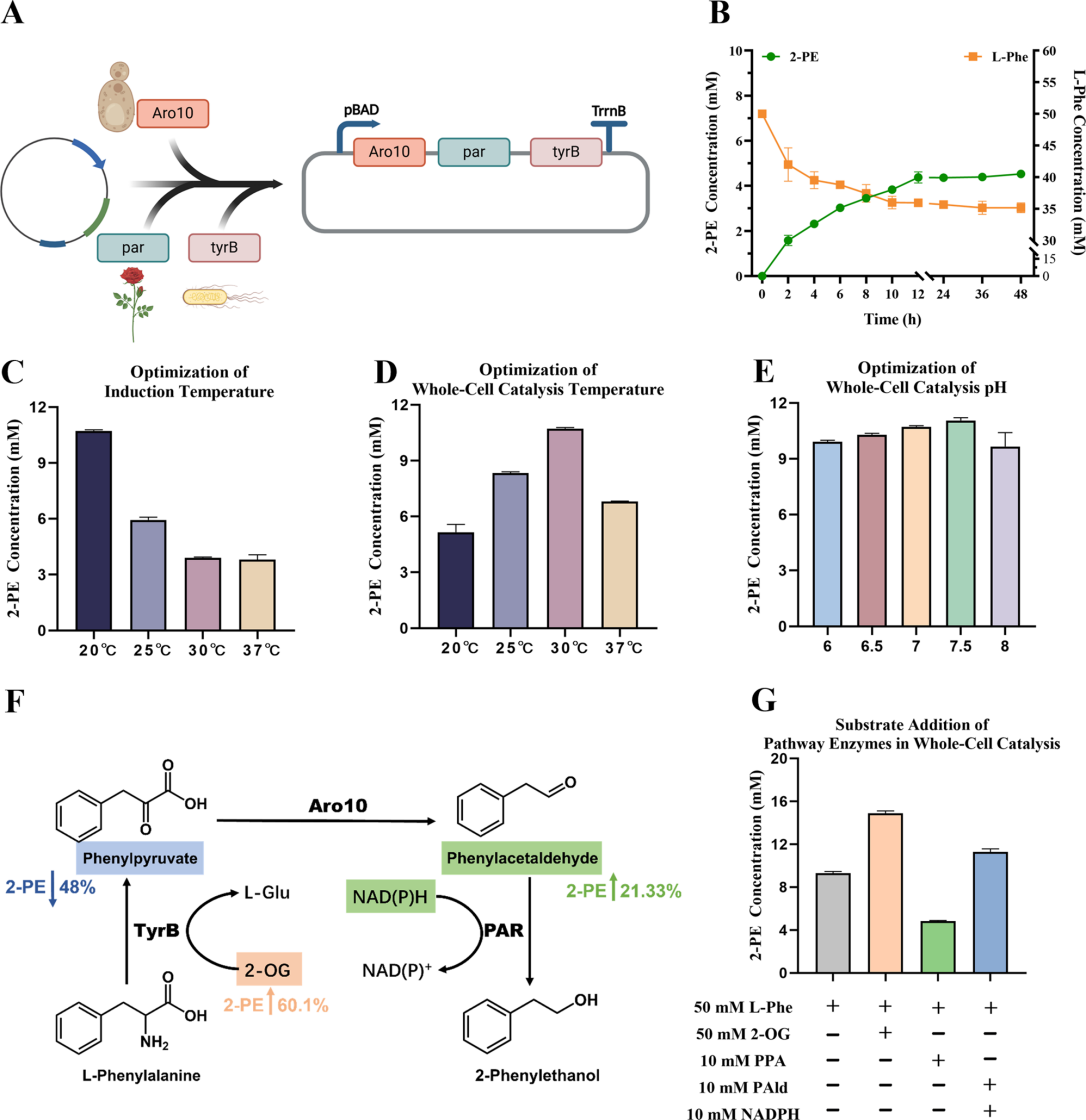
Construction of a 2-PE biosynthetic pathway in *E. coli* and identification of the rate-limiting step. **(A)** Source organisms of the three key enzymes TyrB, Aro10, and PAR involved in the Ehrlich pathway, and modular expression design in plasmid pYB1s-Aro10-PAR-TyrB. **(B)** Time-course of 2-PE production from L-Phe by strain PE01G under small-scale whole-cell biocatalysis conditions. **(C–E)** Effects of induction temperature (**C**), whole-cell biocatalysis temperature (**D**), and reaction pH (**E**) on 2-PE production under small-scale whole-cell biocatalysis conditions. **(F)** Schematic representation of the enzymatic steps in the Ehrlich pathway and the strategy for identifying the rate-limiting step. **(G)** Evaluation of enzymatic efficiencies through the addition of 2-OG, PPA, PAld, and NADPH. Results indicate that Aro10 is the rate-limiting enzyme in this pathway. Data are presented as mean ± standard deviation (*n* = 3)

Building on our previously established GDH-enabled cofactor self-sufficient *E. coli* biocatalyst [71], we first constructed a baseline Ehrlich pathway module in a ΔgdhA chassis to identify the remaining catalytic bottlenecks before reintroducing cofactor regeneration. Specifically, we co-expressed an aromatic amino acid aminotransferase from *E. coli* (TyrB, NCBI: WP032305522.1), a PDC from *Saccharomyces cerevisiae* (Aro10, NCBI: NP010668.3), and a phenylacetaldehyde reductase from *Rosa* species (PAR, NCBI: BAG13450.2), and assembled them into the plasmid pYB1s-Aro10-PAR-TyrB (Fig. 1A), which was transformed into competent *E. coli* BW25113-ΔgdhA cells to obtain the strain PE01G. SDS-PAGE analysis of PE01G after induction at 30 °C revealed that all three enzymes were expressed in soluble form, although Aro10 was partially present in inclusion bodies (Figure S2). The induced cells were then subjected to small-scale whole-cell biocatalysis with 50 L-Phe mM as the substrate. During shaking incubation at 30 °C for 48 h, 2-PE production was monitored dynamically, and the concentration plateaued at 4.53 mM after 12 h (Fig. 1B). These results demonstrated that the heterologous three-enzyme system successfully established a 2-PE biosynthetic pathway in *E. coli*, although the overall yield was limited, likely due to insufficient expression or activity of Aro10.

Considering that protein expression conditions and catalytic parameters significantly affect 2-PE biosynthesis, we optimized the induction temperature, whole-cell biocatalysis temperature, and pH. Among four induction temperatures tested, 20 °C resulted in the highest 2-PE yield (10.7 mM; Fig. 1C), significantly higher than other conditions (*p* < 0.05), indicating that lower temperature facilitates soluble expression and enzymatic activity. Optimization of the catalytic temperature revealed that 30 °C was optimal (10.9 mM), while enzyme activity was severely impaired at 20 °C (Fig. 1D). In terms of pH, although overall differences were minor, the yield was slightly higher at pH 7.5, reaching approximately 10 mM (Fig. 1E).

Despite a 2.4-fold improvement in 2-PE yield under optimized conditions, a large amount of L-Phe remained in the reaction system, suggesting potential bottlenecks in the pathway. To further identify potential rate-limiting steps, we supplemented the small-scale whole-cell biocatalysis system with 2-OG, PPA, and phenylacetaldehyde (PAld) combined with NADPH as diagnostic probes during whole-cell biocatalysis (Fig. 1F). Because several intermediates along the L-Phe-to-2-PE route are difficult to quantify reliably under a single HPLC condition, we used intermediate supplementation as a practical alternative to probe pathway bottlenecks via changes in 2-PE formation. The addition of 2-OG increased the 2-PE yield by 1.6-fold (*p* < 0.05), suggesting that 2-OG availability, rather than TyrB catalytic capacity, constrained the transamination step—consistent with the rationale for the GDH-based “bridge” reported previously [71]. However, the addition of PPA led to a nearly 50% reduction in yield (*p* < 0.05), indicating that simply increasing the intermediate pool does not relieve the pathway limitation and suggesting that downstream phenylpyruvate decarboxylation and/or PPA-associated perturbation may constrain flux under our whole-cell conditions (Fig. 1G). Given that multiple factors could contribute to this phenotype, we treated the PPA-feeding result as qualitative evidence and subsequently verified the dominant role of the decarboxylation step by directly comparing decarboxylases under otherwise identical conditions in the following section.

### Mining and screening of phenylpyruvate decarboxylases identify KivD as the optimal decarboxylase in *E. coli*

Due to the limited soluble expression and catalytic capacity of Aro10 in *E. coli*, and the PPA-feeding phenotype suggesting that phenylpyruvate decarboxylation may constrain flux under our whole-cell conditions, we sought alternative PDCs from other organisms. A phylogeny-guided mining of public databases yielded six representative putative PDCs for experimental screening (Figure S3). The sequences clustered into three major clades, from which we selected six representative enzymes—Aro10, AbPDC, KivD, EkipdC, EaipdC, and PDC_1—for experimental evaluation based on phylogenetic coverage and literature precedents. Their heterologous expression in *E. coli* was preliminarily assessed by SDS–PAGE under identical induction conditions (Figure S4).

**Fig. 2:**
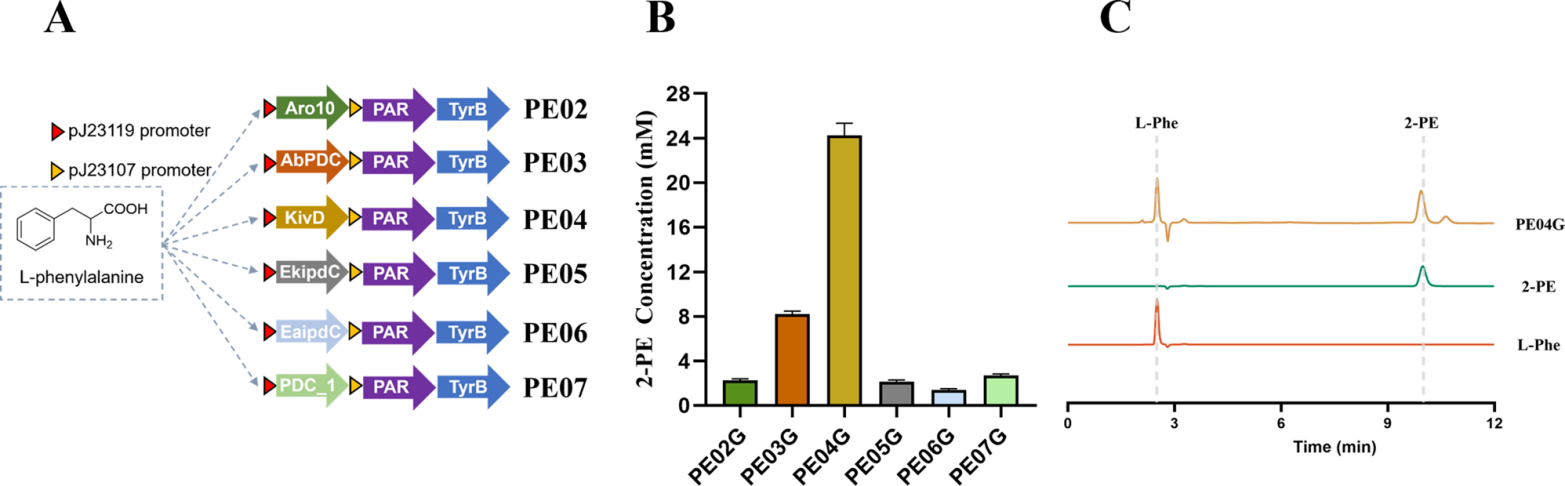
Construction of differential promoter-driven co-expression systems and screening of efficient PDC candidates. **(A)** Design of co-expression modules driven by Anderson promoters. The strong promoter pJ23119 was used to drive the expression of PDC genes, while the medium-strength promoter pJ23107 controlled the expression of PAR and TyrB. **(B)** Comparison of 2-PE production by engineered *E. coli* strains (PE02G–PE07G) expressing different PDC enzymes under small-scale whole-cell biocatalysis conditions. Error bars represent standard deviations from three biological replicates (*n* = 3). **(C)** Representative endpoint HPLC chromatograms of the PE04G whole-cell bioconversion sample after 12 h, showing residual L-Phe and produced 2-PE. The top trace corresponds to the PE04G reaction supernatant, while the middle and bottom traces are pure standards of 2-PE and L-Phe, respectively

To identify the most suitable PDC for the engineered *E. coli* chassis, we evaluated each candidate directly in whole-cell bioconversion and used 2-PE formation as the functional readout. To further improve PDC expression, we designed a co-expression system based on the Anderson promoter library with differential expression strengths. In this construct, the strong promoter pJ23119 was used to drive the expression of PDCs, while the medium-strength promoter pJ23107 was employed to co-express the downstream enzymes PAR and TyrB (Fig. 2A). Plasmids encoding different PDC genes, including Aro10, AbPDC, KivD, EkipdC, EaipdC, and PDC_1, were introduced into *E. coli* BW25113-ΔgdhA to generate engineered strains PE02G to PE07G. Under the previously optimized small-scale whole-cell biocatalysis conditions, the engineered strains were induced and assessed for their ability to convert 50 mM L-Phe to 2-PE after 12 h of bioconversion. As shown in Fig. 2B, significant differences in 2-PE production were observed among strains expressing different PDCs. Notably, strain PE04G harboring KivD exhibited the highest 2-PE titer, reaching 24.26 mM, which was significantly higher than that of PE02G expressing Aro10 (2.24 mM) and all other candidate enzymes (*p* < 0.05), representing a 2.23-fold improvement over the original PE01G strain. HPLC analysis further confirmed substrate consumption and product formation in PE04G, which consumed 26.14 mM of L-Phe and produced 24.26 mM of 2-PE, corresponding to a conversion efficiency of 92.8% (Fig. 2C).

According to the previous phylogenetic analysis, KivD and EkipdC belong to the same evolutionary clade; however, their catalytic performances differed significantly, with KivD substantially outperforming EkipdC. Similarly, AbPDC and EaipdC fall within the same clade, yet AbPDC showed a moderate conversion level (8.14 mM), whereas EaipdC exhibited much lower productivity (1.42 mM). Interestingly, PDC_1, which shares the same clade as Aro10, also displayed comparable performance to Aro10 (2.75 mM vs. 2.24 mM). These results suggest that while phylogenetic proximity may offer preliminary insights into enzymatic potential, actual expression levels and catalytic activities must be experimentally validated. Taken together, considering both catalytic efficiency and expression performance, strain PE04G (KivD) was selected for subsequent studies. With the decarboxylation module substantially improved, we next revisited the GDH-based cofactor regeneration strategy established in our previous work to further enhance whole-cell 2-PE biosynthesis.

### Cofactor regeneration system enhances whole-cell biocatalysis efficiency for 2-PE biosynthesis

**Fig. 3:**
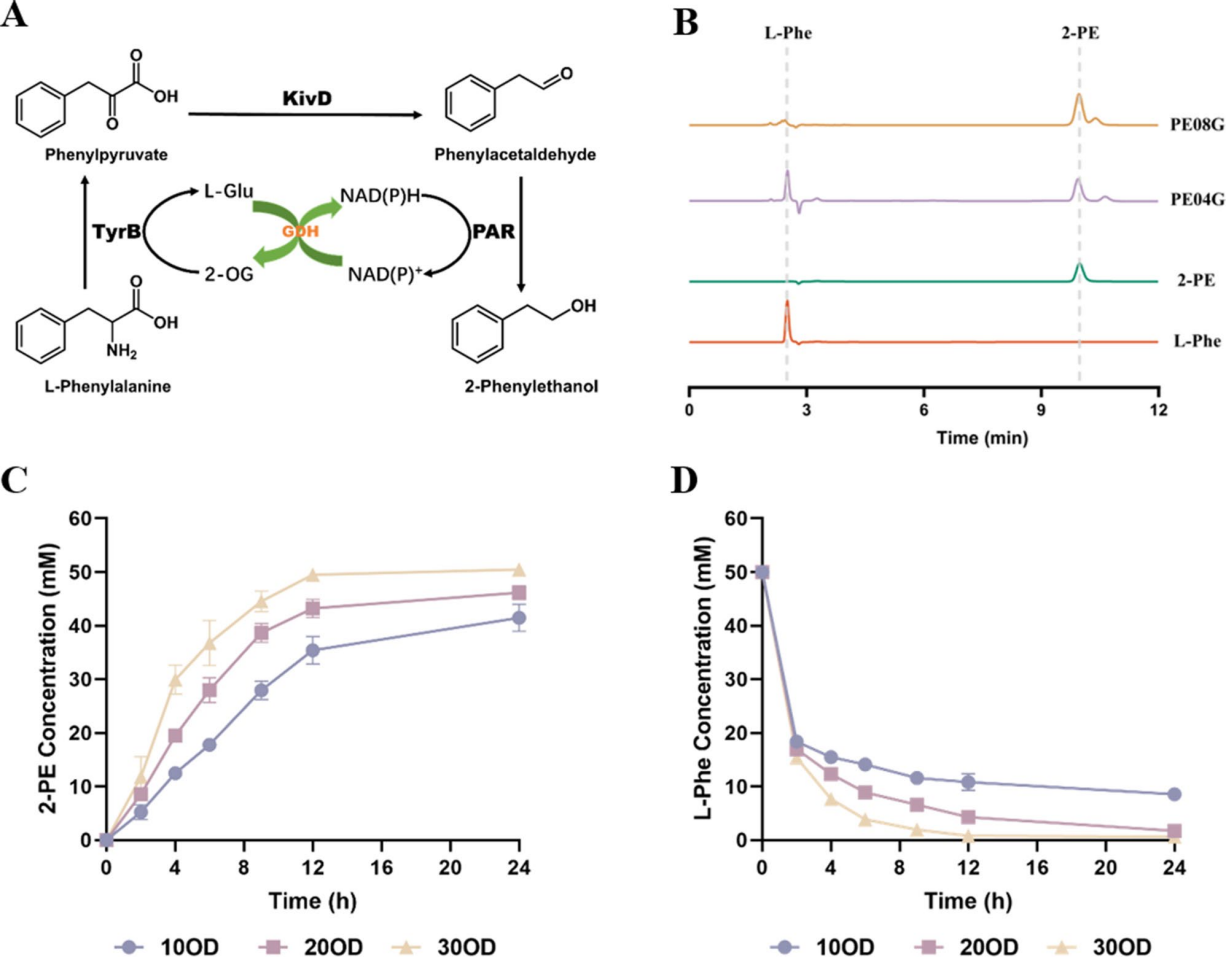
Enhancement of 2-PE production by introducing a cofactor regeneration system. **(A)** Schematic representation of the engineered Ehrlich pathway incorporating the NAD(P)H regeneration module via endogenous GDH activity. **(B)** Representative endpoint HPLC chromatograms of reaction supernatants from PE08G (with GDH-based cofactor regeneration) and PE04G (without cofactor regeneration) after 12 h small-scale whole-cell bioconversion with 50 mM L-Phe, together with pure standards of 2-PE and L-Phe for peak assignment. The traces from top to bottom correspond to PE08G, PE04G, 2-PE standard, and L-Phe standard, respectively. **(C)** Time-course of 2-PE production by strain PE08G at different cell densities (OD₆₀₀ = 10, 20, and 30). **(D)** Corresponding time-course of L-Phe consumption. Data are presented as mean ± SD (*n* = 3)

To evaluate whether the GDH-based cofactor regeneration strategy reported previously [71] could further improve 2-PE production after relieving the decarboxylation bottleneck, we transformed the PE04 plasmid into *E. coli* BW25113 (*gdhA*^*+*^), leveraging the chromosomally encoded gdhA for NADPH regeneration, generating strain PE08G (Fig. 3A). Whole-cell biocatalysis was performed using PE08G, and the parental strain PE04G constructed in BW25113-ΔgdhA served as the control. Under the optimized small-scale whole-cell bioconversion conditions (50 mM L-Phe) and after 12 h of incubation, representative endpoint chromatograms are shown in Fig. 3B. PE08G achieved significantly higher 2-PE production compared to PE04G (*p* < 0.05), reaching a final concentration of 49.5 mM, equivalent to approximately 6.04 g/L. This represents a 2.04-fold increase over the control strain. HPLC analysis confirmed that nearly all of the 50 mM L-Phe was converted in the PE08G system, with a calculated molar conversion efficiency of 99.0%. These results indicate that the cofactor regeneration system facilitated intracellular NADPH supply, supported 2-OG recycling, and maintained redox balance, thereby improving the catalytic efficiency and overall yield of 2-PE.

Because whole-cell biocatalysis is sensitive to biocatalyst loading, which can directly impact reaction rate and space–time yield and may show diminishing returns at higher loadings [73], we evaluated PE08G over a practical range of cell densities. To further characterize the dynamics of substrate utilization and product formation, we analyzed the time-course of whole-cell biocatalysis using 50 mM L-Phe as the substrate with varying cell densities (OD₆₀₀ = 10, 20, and 30; ≈ 3.0, 6.0, and 9.0 g DCW/L, estimated). Samples were collected at 2, 6, 9, 12, and 24 h for quantitative analysis. As shown in Figs. 3C and D, at the highest cell density (OD₆₀₀ = 30; ≈ 9.0 g DCW/L, estimated), strain PE08G almost completely consumed L-Phe within 9 h and reached its maximum 2-PE yield at the same timepoint, with a conversion efficiency of 89.3%. Increasing cell density accelerated both 2-PE accumulation and L-Phe depletion, shortening the time required to approach the endpoint and improving volumetric productivity within the tested range. Therefore, OD₆₀₀ = 30 (9.0 g DCW/L, estimated) was selected as the standard cell loading for subsequent shake-flask studies as a practical balance between rapid conversion and biomass usage.

Collectively, the stepwise optimizations—including whole-cell biocatalysis conditions, decarboxylase selection, co-expression tuning, and reactivation of the GDH-based cofactor regeneration module—led to a significant improvement in 2-PE biosynthesis. The final engineered strain PE08G achieved a peak 2-PE titer of 49.5 mM, representing a 10.9-fold increase compared to the initial strain PE01G.

### Enhancing flask-scale 2-PE production via in situ extraction strategy

**Fig. 4:**
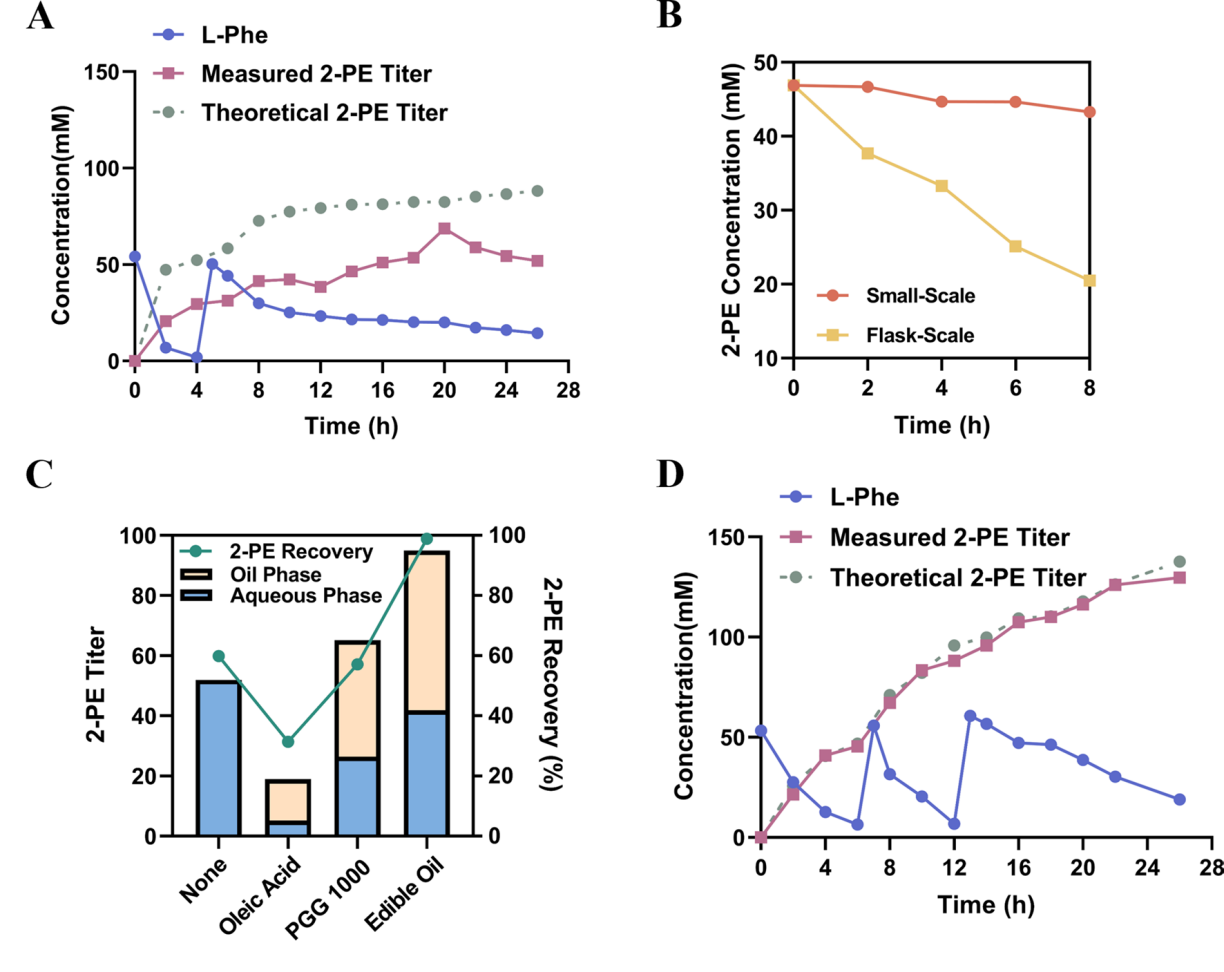
Evaluation of the in situ extraction strategy for enhancing flask-scale 2-PE biosynthesis. **(A)** Time-course profiles of L-Phe consumption and 2-PE production under flask-scale whole-cell biocatalysis using a fed-batch strategy at RSM-predicted optimal conditions. Theoretical 2-PE titers were calculated based on complete substrate-to-product conversion. **(B)** Comparison of 2-PE volatilization between small-scale and flask-scale systems, based on residual 2-PE concentrations after shaking incubation. **(C)** Effects of different in situ extractants, including oleic acid, PPG 1000, and edible oil, on 2-PE titer and recovery. Total 2-PE recovery includes both aqueous and oil phases. **(D)** Time-course profiles of L-Phe consumption and 2-PE accumulation when the volume ratio of edible oil to reaction solution was adjusted to 2:1. The comparison between theoretical and actual titers indicates high conversion efficiency

To improve 2-PE recovery and alleviate product-associated inhibition during shake-flask whole-cell biocatalysis, we scaled up the biocatalysis system to the flask level. We first employed RSM to optimize critical parameters, including induction temperature (15–40 °C), whole-cell biocatalysis temperature (15–40 °C), and pH (5–9) (Figure S5). According to model predictions, the optimal conditions were as follows: induction temperature 29.01 °C, catalysis temperature 28.67 °C, and pH 7.12. Under these conditions, flask-scale reactions were initiated with 50 mM L-Phe as the initial substrate. Given the known inhibitory effect of high L-Phe concentrations, a fed-batch strategy was applied: substrate levels were monitored every 2 h, and L-Phe was replenished to 50 mM when depleted. As shown in Fig. 4A, L-Phe was rapidly depleted within the first 4 h, and a high conversion rate was maintained between 4 and 8 h, after which the reaction rate gradually declined. By 26 h, a total of 88.2 mM L-Phe had been consumed. Although complete conversion of L-Phe to 2-PE was theoretically possible, only 52.0 mM of 2-PE was detected, suggesting substantial product loss. We hypothesized that this discrepancy resulted from 2-PE volatilization during the reaction. To test this, we compared 2-PE evaporation under small-scale and flask-scale conditions. With an initial 2-PE concentration of 46.9 mM, only 7.70% was lost in the small-scale system after 8 h of shaking, whereas the loss reached 56.3% in the flask-scale system (Fig. 4B), indicating that significant volatilization occurs under higher aeration conditions if no collection method is applied.

To mitigate product loss and reduce 2-PE toxicity toward cells, we introduced an in situ extraction strategy by supplementing the reaction system with different extraction solvents. Based on previous studies, oleic acid and PPG were selected and added to the reaction mixture at a 1:1 (v/v) ratio in 250 mL shake flasks. Experimental results showed that oleic acid performed poorly: L-Phe consumption dropped to 60.4 mM (a 31.6% decrease compared to the no-extraction control), and the total 2-PE yield (from both aqueous and oil phases) was only 31.3%, representing a 28.5% reduction. In contrast, PPG 1000 significantly enhanced L-Phe consumption to 114 mM (a 22.6% increase over the control), and the total recovered 2-PE reached 65.1 mM, 13.1 mM higher than the no-extraction group. However, the overall recovery rate remained modest at 57.1%, indicating considerable product loss. Considering the appreciable volatility of 2-PE under vigorous shaking and the partitioning of 2-PE into a hydrophobic phase, we further tested food-grade soybean oil as the extraction phase. Phase separation with soybean oil was more distinct than that with oleic acid or PPG (Figure S6). When used at a 1:1 (v/v) ratio, L-Phe consumption reached 96.1 mM, slightly lower than with PPG, but the total 2-PE recovered from both phases was 95.0 mM, with a remarkably high recovery efficiency of 98.9% (Fig. 4C).

We speculated that under the 1:1 ratio, some 2-PE remained in the aqueous phase, potentially accumulating to toxic levels and inhibiting further substrate conversion. Therefore, we increased the soybean oil-to-aqueous ratio to 2:1. The resulting bioconversion showed a substantially delayed decline in reaction rate. A high production rate was maintained during the first 12 h (approximately 7.99 mM/h), and after the third substrate addition, the rate gradually dropped to 3.09 mM/h. After 26 h, 138 mM L-Phe was consumed (30.2% higher than the 1:1 group). The total 2-PE titer reached 130 mM (15.9 g/L), calculated on an aqueous-volume basis from the combined 2-PE quantified in the aqueous and oil phases. The overall recovery was 94.2% relative to the theoretical 2-PE estimated from L-Phe consumption (Fig. 4D). Based on the final 2-PE titer of 15.9 g/L achieved after 26 h, the average volumetric productivity was 0.61 g 2-PE/L·h, and the average specific productivity was 0.020 g 2-PE/g DCW·h using the OD_600_-based DCW estimation described above.

## Discussion

Building on our previously reported cofactor self-sufficient *E. coli* whole-cell platform for Ehrlich-type 2-PE bioconversion [71], we achieved high-level whole-cell bioconversion of L-Phe to 2-PE by systematically constructing and optimizing the Ehrlich pathway. Notably, although the GDH-based bridge was stoichiometrically designed to balance 2-OG and NAD(P)H recycling, our previous platform did not reach quantitative conversion, underscoring that relieving kinetic bottlenecks is essential for translating cofactor self-sufficiency into near-complete substrate conversion. Accordingly, we implemented a stepwise optimization workflow that first used whole-cell substrate-feeding assays to diagnose the remaining bottleneck and pinpoint phenylpyruvate decarboxylation as rate-limiting, then restored decarboxylation flux via phylogeny-guided enzyme substitution and expression tuning, and finally coupled the upgraded pathway with GDH-supported cofactor recycling and a food-grade soybean-oil overlay for biocompatible ISPR. Ultimately, a 2-PE titer of 15.9 g/L was achieved at the shake-flask level, representing, to our knowledge, the highest 2-PE titer reported for Ehrlich-based production in *E. coli*, and notably achieved at this scale. Our shake-flask performance closes the gap between genetically tractable *E. coli* chassis and high-titer systems that often rely on non-conventional yeast hosts and specialized ISPR modules [55, 59, 74]. Together, these design elements enabled a platform with high conversion efficiency, elevated yield, and excellent biocompatibility—highlighting its strong potential for scale-up and industrial translation. To benchmark our system against representative Ehrlich-based whole-cell platforms, we summarized reported 2-PE production systems in *E. coli* and yeast hosts (Table 1). Importantly, using *E. coli* as a model and genetically tractable host provides a practical entry point for future integration with established *E. coli* L-Phe overproduction modules, moving toward de novo production from sugars and scalable bioprocess development.

**Table 1:** Representative whole-cell 2-PE production systems based on the Ehrlich pathway from L-Phe in *E. coli* and yeast hosts

Host	Substrate	ISPR strategy	2-PE titer	Scale	Key features	Reference
*S. cerevisiae* BD	L-Phe + sucrose	Resin ISPR (D101 macroporous resin)	6.17 g/L	250 mL shake flask	D101 macroporous resin adsorbs ~ 50% of 2-PE, giving a molar yield > 0.70 from L-Phe	[68]
*K. marxianus* CBS 600	L-Phe + glucose	Polymer TPPB (Hytrel beads)	20.4 g/L	3 L bioreactor	External, replaceable Hytrel columns sequester 2-PE and relieve product inhibition	[76]
*S. cerevisiae* Ye9-612	L-Phe + glucose	Solid–liquid ISPR (PMMA microspheres)	7.05 g/L	3 L bioreactor	Swellable PMMA microspheres encapsulate ~ 76% of 2-PE with high uptake per gram	[69]
*S. cerevisiae* JM2014	L-Phe + glucose + sucrose	Biphasic oil overlay (rapeseed oil)	9.79 g/L	4.5 L bioreactor	Food-grade rapeseed oil extracts 2-PE while serving as an auxiliary carbon source and rose-scented co-product	[62]
*E. coli* BW25113	L-Phe	Polymer TPPB (Hytrel beads)	9.14 g/L	5 L bioreactor	GDH-based cofactor self-sufficiency combined with zeolite-assisted NH₄⁺ removal to drive 2-PE formation	[73]
*P. fermentans WUT36*	L-Phe + corn stover hydrolysate	No ISPR	3.66 g/L	1 L shake flask	Corn-stover hydrolysate supplies low-cost C5/C6 sugars for 2-PE in a complete biomass-to-product workflow	[61]
*Y. lipolytica* CH 1/5	L-Phe + Crude glycerol	No ISPR	3.2 g/L	3.7 L bioreactor	Step-wise L-Phe fed-batch on crude glycerol improves 2-PE titer and L-Phe conversion	[57]
*Acinetobacter soli* ANG344B	L-Phe + glucose	ISPA (Amberlite XAD 4 resin)	6.99 g/L	2 L bioreactor	Amberlite XAD 4 adsorption increases 2-PE titer ~ 3.3-fold and exploits the resin’s high capacity	[70]
*S. cerevisiae* D-22	L-Phe + glucose	Biphasic oil overlay (oleic acid)	6.41 g/L	5 L bioreactor	Oleic-acid overlay both extracts 2-PE and stabilizes membranes, mitigating toxicity and improving L-Phe conversion	[71]
*E. coli* BW25113	L-Phe	Biphasic oil overlay (soybean oil)	15.9 g/L	250 mL Shake flask	KivD engineering relieves the decarboxylation bottleneck, enabling high L-Phe conversion with soybean-oil overlay	This work

In our initial Aro10-based *E. coli* construct, substrate-feeding tests suggested that phenylpyruvate decarboxylation was rate limiting for 2-PE formation, implicating Aro10 as the major bottleneck enzyme. However, Aro10 showed limited soluble expression in *E. coli* (Figure S2), and the SDS-PAGE comparison across candidate PDCs further highlighted substantial differences in solubility and expression (Figure S4). To overcome this limitation, we developed a phylogeny-guided screening strategy for PDCs, curating 33 PDC homologs and prioritizing six representative enzymes for experimental evaluation using a differential-promoter co-expression design. Experimental results demonstrated that KivD from *Lactococcus lactis* significantly outperformed Aro10 in our *E. coli* system, markedly improving 2-PE formation, and thus represented a more suitable PDC for Ehrlich-type bioconversion in *E. coli*. Notably, although KivD clustered with several less active enzymes, it showed superior expression and activity in our system, underscoring that phylogenetic proximity alone does not guarantee performance. Nevertheless, phylogenetic analysis remains valuable for prioritizing diverse candidates and reducing the screening burden when coupled with experimental validation.

Once the decarboxylation step was accelerated, the cofactor-recycling module could be kinetically aligned with aldehyde formation, allowing recycled NAD(P)H to be efficiently consumed in the downstream reduction. To address this issue, we reintroduced a GDH-enabled redox self-cycling module by harnessing the chromosomally encoded GDH in *E. coli* to reinforce intracellular NAD(P)H regeneration. With the upstream decarboxylation bottleneck relieved, the GDH-enabled module provided a sustained NAD(P)H supply for PAld reduction, boosting 2-PE accumulation and enabling near-quantitative L-Phe conversion (98.94%). This result suggests that the benefit of GDH-assisted redox recycling is maximized once the upstream decarboxylation flux is restored.

As a lipophilic aromatic alcohol, 2-PE is prone to volatilization losses during fermentation and exerts cytotoxic effects on microbial hosts, severely limiting its accumulation in the reaction system [64, 67, 75]. To address these challenges, we implemented an ISPR strategy by establishing biphasic systems that continuously partition 2-PE into a non-aqueous phase, thereby reducing both aqueous-phase toxicity and volatilization losses under aerated shake-flask conditions. Earlier Ehrlich-based whole-cell platforms have often relied on tailored ISPR formats to mitigate 2-PE inhibition (Table 1), motivating us to explore an operationally simpler and food-compatible overlay for routine shake-flask production. Comparative evaluations of conventional extractants—oleic acid and PPG—revealed limitations in extraction efficiency and potential inhibitory effects on cell viability. In contrast, edible oils not only formed well-defined biphasic layers with the aqueous phase but also demonstrated excellent biocompatibility, significantly enhancing 2-PE recovery without impairing microbial activity [67, 76]. Under optimized conditions, a 2:1 biphasic system using soybean oil achieved a recovery efficiency of up to 94.2%, with a final 2-PE titer of 15.9 g/L. We evaluated soybean-oil overlays at only two oil-to-aqueous ratios (1:1 and 2:1, v/v) to demonstrate feasibility in shake flasks. Future work will expand ratio screening (0.5:1, 1.5:1, and potentially up to 3:1) to balance recovery performance with oil usage and handling. Higher oil fractions may improve extraction capacity, but they also increase extractant consumption and can add mixing and phase-separation burden, which affects cost and sustainability at scale. As noted for two-phase bioprocesses, extractant cost, recyclability, and downstream separation complexity are key determinants of overall feasibility [77]; therefore, ratio selection during scale-up should be guided by techno-economic evaluation. Food-grade soybean oil is inexpensive, readily available, and compatible with flavor applications, offering a practical alternative to conventional organic extractants. Given its high 2-PE partitioning capacity and good biocompatibility, this overlay strategy should be readily adaptable to other volatile or inhibitory aromatic alcohols and related products.

We acknowledge that the performance of an oil-overlay ISPR may change upon scale-up, as hydrodynamics and gas–liquid–oil mass transfer differ between shake flasks and stirred bioreactors. While increased agitation and aeration can enhance interfacial area and extraction, they may also increase emulsification, hinder phase separation, and alter oxygen transfer; accordingly, the oil-to-aqueous ratio and mixing conditions will require re-optimization at larger scale [78, 79]. We also note that whole-cell immobilization is a viable alternative to facilitate product removal and enable catalyst reuse, but it can introduce diffusion limitations and additional process complexity. Given the scope of this study, we focused on the food-grade oil-overlay strategy, and future work will evaluate immobilized formats alongside oil-based ISPR and compare productivity, operational stability, and downstream separability.

Scale-up from aerated shake flasks to mechanically stirred bioreactors (5–100 L) may change 2-PE losses: a lower surface-area-to-volume ratio can reduce evaporation from the liquid surface, whereas increased aeration and agitation can enhance stripping of volatile 2-PE into the off-gas [80]. Two-phase systems may also exhibit greater oil dispersion and partial emulsification under higher shear, which can hinder phase separation and complicate sampling and downstream recovery; this can be mitigated by using lower-shear impellers, operating at lower tip speeds, and implementing defined settling or coalescence steps before sampling or phase recovery [81]. No preliminary 5–100 L bioreactor experiments were performed in this study.

Although our platform enables efficient L-Phe-to-2-PE bioconversion, reliance on exogenous L-Phe remains a major cost and sustainability constraint. Moving toward de novo production from sugars will require strengthening aromatic amino acid precursor supply and relieving feedback regulation within the shikimate and L-Phe network [82, 83], so that flux can be effectively delivered into the optimized Ehrlich module. In parallel, the Ehrlich route is readily compatible with renewable and waste-derived feedstocks, offering a near-term path to reduce raw-material inputs while maintaining high conversion. Accordingly, the modular *E. coli* whole-cell chassis established here provides a practical starting point for both sugar-based de novo synthesis and waste-valorization bioprocess development.

## Conclusions

Building on our previously reported cofactor self-sufficient *E. coli* whole-cell platform, we optimized the Ehrlich module for efficient conversion of L-Phe to 2-PE by sequentially addressing the main bottlenecks in decarboxylation, redox supply, and product loss. We identified phenylpyruvate decarboxylation as the dominant bottleneck and relieved this constraint by replacing Aro10 with KivD. With the decarboxylation step improved, re-introducing the GDH-based cofactor recycling module strengthened the reduction capacity and improved conversion performance under whole-cell conditions. In addition, optimizing biocatalyst loading and substrate feeding supported rapid conversion in the shake-flask process. A food-grade soybean-oil overlay provided biocompatible in situ recovery during shake-flask bioconversion, mitigating toxicity and volatilization losses, and supported repeated feeding, resulting in 15.9 g/L 2-PE. This upgraded *E. coli* platform provides a practical basis for coupling with established L-Phe overproduction modules and for further process intensification toward de novo synthesis and scale-up.

## Supplementary Information

Below is the link to the electronic supplementary material.

Supplementary Material 1

## Data Availability

The datasets used and analysed during the current study are available from the corresponding author on reasonable request.

## Abbreviations

2-PE: 2-phenylethanol
BBD: Box–Behnken design
GDH: Glutamate dehydrogenase
HPLC: High-performance liquid chromatography
ISPR: In situ product recovery
KivD: Alpha-ketoisovalerate decarboxylase
L-Phe: L-phenylalanine
NAD(P)H: Nicotinamide adenine dinucleotide (phosphate), reduced form
OD_600_: Optical density at 600 nm
PAld: Phenylacetaldehyde
PAR: Phenylacetaldehyde reductase
2-OG: 2-oxoglutarate
PDC: Phenylpyruvate decarboxylase
PPA: Phenylpyruvate
PPG: Polypropylene glycol
RSM: Response surface methodology
SDS-PAGE: Sodium dodecyl sulfate–polyacrylamide gel electrophoresis
