# Supplementary material for: High-titer 2-phenylethanol production in Escherichia coli via decarboxylation-step optimization and a vegetable-oil overlay for biocompatible in situ recovery
Source: J Biol Eng. 2026 Mar 27;20:84. doi: 10.1186/s13036-026-00667-4 (PMC13147604; doi:10.1186/s13036-026-00667-4)
Supplement: Supplementary file 1 — Supplementary Material 1 [file 13036_2026_667_MOESM1_ESM.docx]

**High-titer 2-phenylethanol production in *Escherichia coli* via decarboxylation-step optimization and a vegetable-oil overlay for biocompatible in situ recovery**

**Supplementary material**

**Supplementary tables and figures**

**Table S1. Engineered *E. coli* strains constructed and used in this study.**

| **Strain** | **Plasmid(s)** | **Host strain** | **Source** |
| --- | --- | --- | --- |
| BW25113 | - | BW25113 | Commercial source |
| BW-ΔgdhA | - | BW25113-ΔgdhA | This Lab |
| PE01G | PE01 (pYB1a-Aro10-par-tyrB) | BW-ΔgdhA | This study |
| PE02G | PE02 (pY97A-Aro10-par-tyrB) | BW-ΔgdhA | This study |
| PE03G | PE03 (pY97A-AbPDC-par-tyrB) | BW-ΔgdhA | This study |
| PE04G | PE04 (pY97A-KivD-par-tyrB) | BW-ΔgdhA | This study |
| PE05G | PE05 (pY97A-EkiPDC-par-tyrB) | BW-ΔgdhA | This study |
| PE06G | PE06 (pY97A-EaiPDC-par-tyrB) | BW-ΔgdhA | This study |
| PE07G | PE07 (pY97A-PDC_1-par-tyrB) | BW-ΔgdhA | This study |
| PE08G | PE04 (pY97A-KivD-par-tyrB) | BW25113 | This study |

All plasmids used in this study carried an ampicillin-resistance marker (AmpR); ampicillin was used at 100 mg/L unless otherwise stated.

**
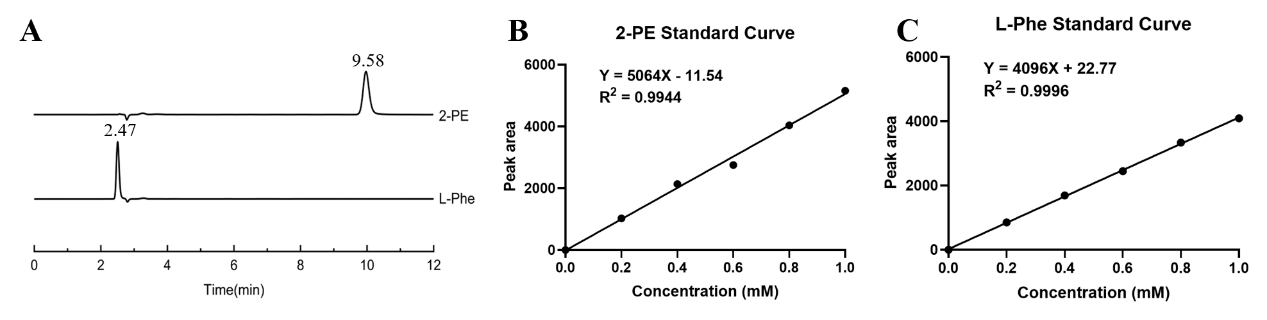
**

**Figure S1. HPLC chromatograms and standard curves for analytical-grade L-Phe and 2-PE.** **(A)** HPLC chromatograms of L-Phe and 2-PE standards showing retention times of 2.47 min and 9.58 min, respectively. **(B)** Standard curve for 2-PE (Y = 5064X – 11.54, R² = 0.9944). **(C)** Standard curve for L-Phe (Y = 4096X + 22.77, R² = 0.9996).

**
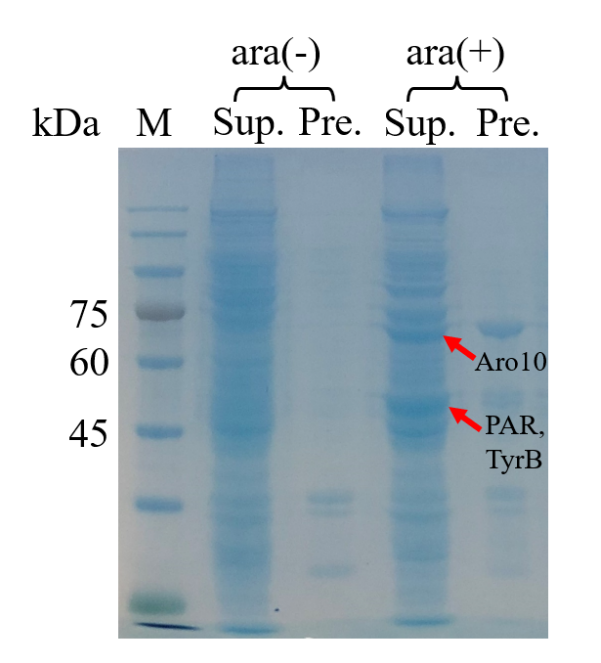
**

**Figure S2. SDS-PAGE analysis of protein expression in strain PE01G with and without L-arabinose induction.** Cells were cultivated in ZYM-5052 medium and analyzed for soluble (Sup.) and insoluble (Pre.) fractions. M: protein marker; ara(−): uninduced condition; ara(+): induced with 0.2% (w/v) L-arabinose. Distinct bands corresponding to Aro10 and PAR/TyrB are indicated by red arrows.


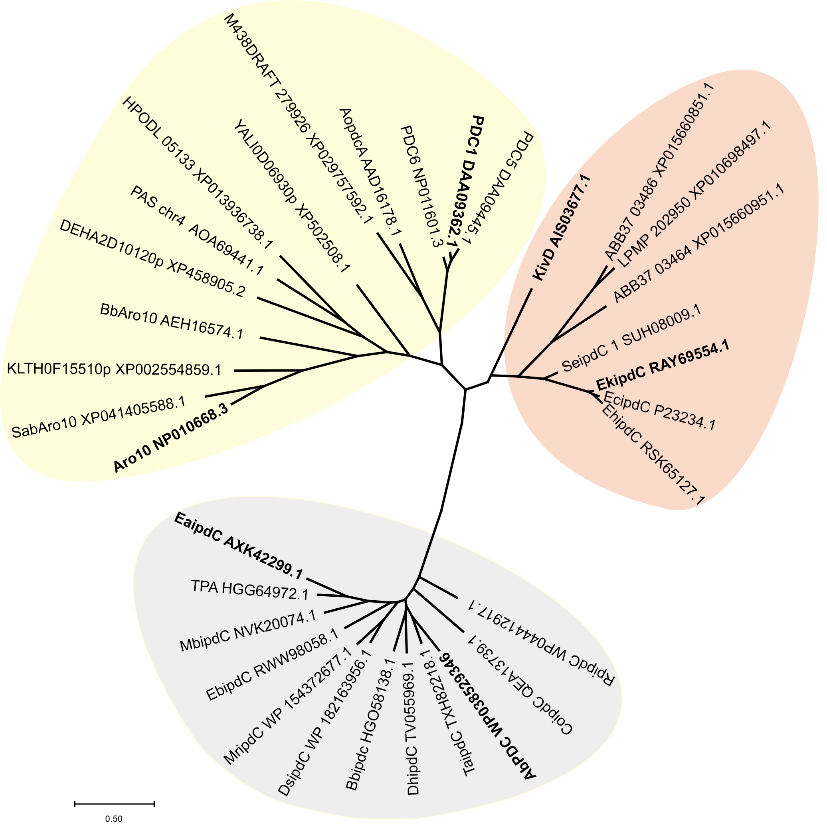


**Figure S3. Phylogenetic analysis of 33 PDC candidates from diverse species.** A phylogenetic tree was constructed in MEGA 11 using the NJ method based on amino acid sequences aligned with ClustalW. Genetic distances were calculated using the p-distance model, and bootstrap values were determined from 1000 replicates. The tree reveals three major clades, highlighted in different colors. Six representative enzymes (in bold), including Aro10, AbPDC, KivD, EkipdC, EaipdC, and PDC_1, were selected for further functional characterization.


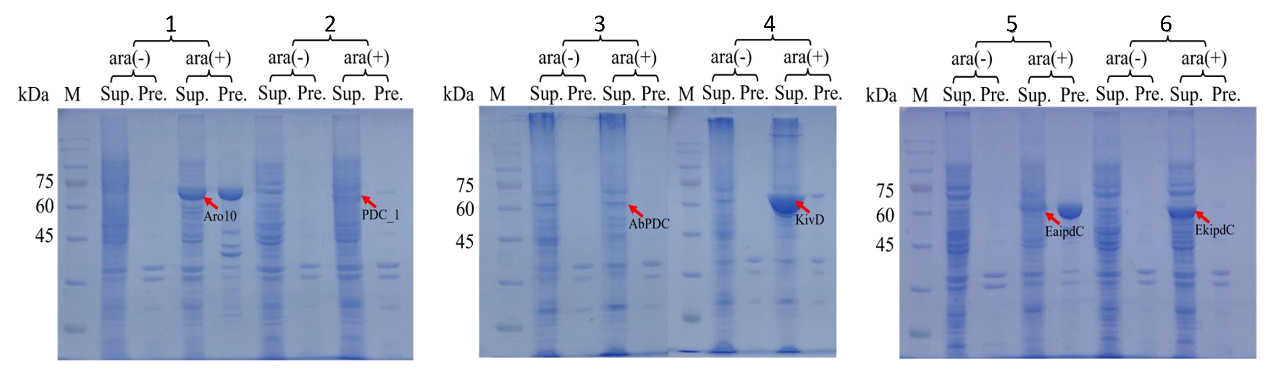


**Figure S4. SDS-PAGE analysis of protein expression for six candidate PDCs in engineered *E. coli* strains under L-arabinose induction.** Cells were cultivated in ZYM-5052 medium and analyzed for soluble (Sup.) and insoluble (Pre.) fractions. M: protein marker; ara(−): uninduced condition; ara(+): induced with 0.2% (w/v) L-arabinose. Lanes 1–6 correspond to Aro10, PDC_1, AbPDC, KivD, EaipdC, and EkipdC, respectively, and the expected PDC bands are indicated by red arrows.

**
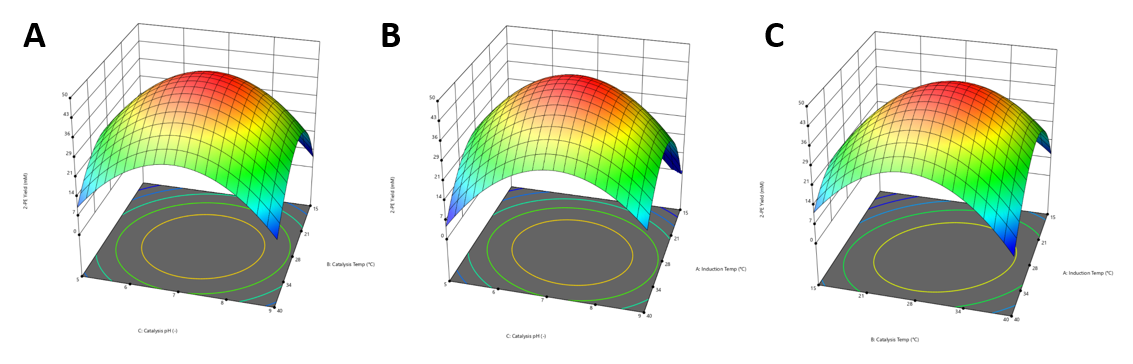
**

**Figure S5. Response surface analysis of 2-PE production as affected by induction temperature (X₁), catalytic temperature (X₂), and catalytic pH (X₃). (A)** Interaction between catalytic temperature and pH; **(B)** interaction between induction temperature and catalytic pH; **(C)** interaction between induction temperature and catalytic temperature. Surface plots were generated using a Box–Behnken Design in Design-Expert software.


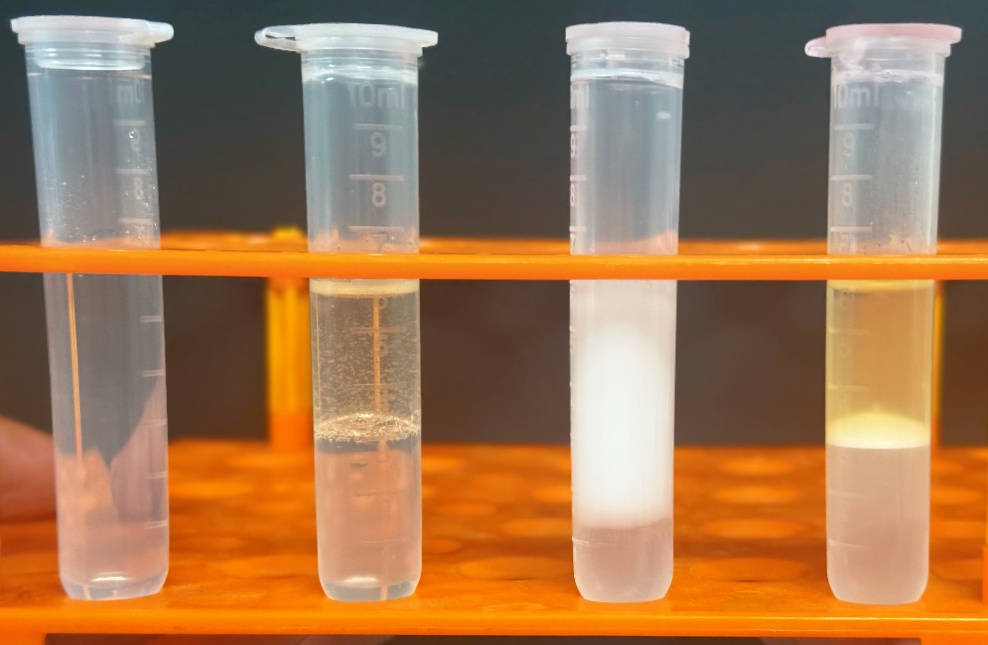


**Figure S6. Phase separation behavior of different in situ extraction systems used in 2-PE biosynthesis.** From left to right: aqueous phase only (no extractant), edible vegetable oil, PPG 1000, and oleic acid. Clear bilayer formation is observed for all extractants except the aqueous-only control, with vegetable oil showing sharp phase clarity and minimal emulsion formation.
